# Staff retention after the privatization of township-village health centers: a case study from the Haimen City of East China

**DOI:** 10.1186/1472-6963-13-136

**Published:** 2013-04-12

**Authors:** Jiayan Huang, Lu Shi, Yingyao Chen

**Affiliations:** 1 Key Laboratory of Public Health Safety, Ministry of Education (Fudan University), Shanghai, China; 2 Department of Public Health Sciences, Clemson University, 525 Edwards Hall, Clemson, SC 29634-0745, USA

**Keywords:** Township health center, Ownership reform, Health professional, Permanent staff

## Abstract

**Background:**

Township-village health centers in rural areas play an important role in health service system in China. In East China’s Jiangsu Province, the City of Haimen privatized all 25 township-village health centers in 2002. This study assesses the effect of privatization on staff retention among these health centers.

**Methods:**

This is a retrospective study based on 10-year administrative data from Haimen City. Three waves of administrative data were collected in 2000 (2 years before privatization), 2005 (3 years after privatization) and 2009 (7 years after privatization) for all health care providers in Haimen City, including 3 county hospitals, 6 central township health centers (CTHC) and 25 township-village health centers (TVHC). The effect of privatization on TVHCs’ staff retention was evaluated in comparison with the other two types of health care providers. We conducted focus groups with people from Haimen Bureau of Health and various health care providers to help understand the context of these administrative statistics.

**Results:**

Each township-village health centers had an average of 40 staff members before the privatization, and the majority of those staff members were their permanent staff. In 2005, three years after the privatization, a substantial amount of staff decrease (from 39.7 staff members per TVHC to 27.5 per TVHC) occurred in these township-village health centers. From 2000 to 2009, the total payroll in TVHCs decreased by almost 29%, while the number of their permanent staff members and nurses decreased by more than 40%. Among the two types of health care providers that did not go through a privatization, those central township health centers had no significant change on their payroll size during this period whereas the county hospitals’ average payroll size actually increased by 20%, especially for the number of doctors. In addition, the average salary and caseload in TVHC showed similar decreasing trends from 2000 to 2009, while no such trends can be observed among the other two types of providers that did not undergo privatization.

**Conclusion:**

The privatization of township-village health center could have adverse effects on their staff retention, a phenomenon that occurs with a decrease in salary and caseload in these centers. To ensure that these health institutions keep providing health care for rural communities, a stronger social safety net and stronger financing of rural health insurance might be helpful in their staff retention.

## Background

Hospital ownership reform is an important topic for health services researchers worldwide [1]. In China, hospital ownership reform began from 1997 and accelerated during the next ten years [2]. The Chinese development is no exception from other countries’ patterns whereby the resource constraints typical of public/nonprofit hospitals contributed to the trend of ownership conversion [3,4]. Below we will briefly describe the background of hospital ownership reform in the mainland China.

As the community level of health delivery system in China, township health centers in rural areas are aimed to provide primary care, short-term emergency care, long-term care, and a few specialized health services [5-7]. Until the end of 2010, there were about 38 thousand township-village health centers in China, most of which are owned by local governments [8]. Compared to community health care centers in urban areas, many township health centers had faced lack of administrative resources, low-level quality of health service, under-regulated medical practice [9-11]. In 2001, the central government published “Strategy on health system reform and development in rural areas” [12], which ruled that county governments take financial responsibility for developing public township health centers. Under this new regulatory environment, rural township health centers had become the pioneer of ownership conversion reform with local governments eagerly privatizing them to avoid the financial responsibility [13-15]. Many government-owned township health centers were then privatized, mainly of which are on nonprofit status. The function of these health centers was focused on basic medical services.

In China’s public hospitals and public health centers, physicians, nurses and hospital technicians are generally salaried permanent staff members. These permanent staff members in public hospitals and public health centers in China enjoy guaranteed benefits from health insurance and retirement pensions. And governments reimburse public institutions the majority of these human resource’ salaries and benefits [16]. In contrast, private health institutions had to pay all of staff members out of their own pocket, from pension to health insurance. This means that those physicians, nurses and technicians in privatized health centers are in a much worse social safety net than their competitors in public hospitals. In an aging society where health care cost and living cost both rise fast, this could mean serious challenges in employee retention for the privatized health centers. Even before the ownership reform, insufficient staffing has been a main drawback influencing public township health center’s development [17]. It is necessary then for researchers to pay attention to the issue of staff retention after the privatization of township health centers.

The impact of ownership reform has been studied from many different angles: quality of care, human resources, hospital capacity, hospital finances, undercompensated care, etc. [18-22]. However, studies of China’s hospital ownership reform are mainly short-term evaluation [23-25]. Moreover, these studies lacked the proper “control” sample of hospitals that had not gone through ownership reform, while the latter were systematically different from those privatized hospitals even before the ownership reform [23].

Based on 10-year panel data, this study tries to assess the effect of ownership conversion on staff retention in township health center performance. We include all of non-converted hospitals in the same city as the control group, and track the performance change from the pre-reform fiscal year to those post-reform fiscal years. This enables us to look at the issue with a more holistic and long-term perspective than previous studies.

## Methods

### Study setting

This is a retrospective study. The site is Haimen City in Jiangsu Province, a coastal province in East China (north of Shanghai). Haimen City included 23 townships and registered 9 million residents in 2010 [26]. Its per capita GDP was 55.6 thousand Yuan and the disposable income per capita among urban residents was 22.9 thousand Yuan in 2010. Child mortality (before Age 5) and maternal mortality were 8.35 per 1000 live birth and 15.79 per 100,000 pregnant women, respectively, in 2007 [27].

Before 2002, each of Haimen’s then 25 townships had one township-village health center (TVHC) to provide primary medical services and public health services. In addition, there were 6 central township health centers (CTHCs) in Haimen. A central township health center generally plays similar functions as those of a TVHC with moderately better equipment and a larger catchment area typically covering several townships. In addition, there were 3 county hospitals in Haimen, who provided more comprehensive health services with more facilities and more human resources than TVHCs and CTHCs.

Both types of township health centers in 2000 and 2001 faced the issue of underfunding from the government. To relieve itself of the fiscal responsibility for health centers, Haimen City Government sold all 25 township-village health centers to the private sector in 2002 [27]. Most buyers of these health centers were former chief executive officers of the same center, and staff members of these newly privatized centers elected their new directors after the privatization. Although the government retained its pension promise for those who stayed in these privatized health centers, their new pension deal is much inferior to that in public hospitals in that this pension benefit could only be paid out when the beneficiary reaches the official retirement age (while the benefit payout is a lot more flexible for those in the public sector). The government maintained the public ownership of central township health centers and county hospitals.

### Qualitative data collection

Our qualitative data collection was conducted between May 2010 and May 2011. Site visits were carried out in three privatized TVHCs and one county hospital. After that, we conducted four focus groups in Haimen, where the informants included directors of Haimen Bureau of Health (HBH), HBH department managers (Department of Medicine and Department of Finance), one county hospital manager, owners from privatized TVHCs, TVHC clinic department heads and practitioners. These focus groups were meant to help us learn more about the process of privatization and to facilitate the interpretation of quantitative data analysis results.

### Administrative data collection

In May 2010, questionnaires were sent to hospital managers to collect administrative information about the health center or hospitals. Variables collected include the service capacity of the provider (number of beds, number of medical devices with a purchase price over 10 thousand Yuan and total asset value), the number of staff members (physicians, nurses, technicians), caseload (annual number of visits, annual number of discharges) and the annual financial figures (the total revenue and average salary).

Some data we used for this study were from official database of Haimen Bureau of Health, and we received its permission to use it.

### Data analysis

The average payroll size of the three types of health care providers were first measured at the baseline two years before the privatization (2000), then 3 years (2005) and 7 years (2009) after the 2002 privatization. Then we ran one-way ANOVA tests for each type of health care providers to examine whether the average payroll size, average number of physicians, average number of nurses and average number of permanent staff members were significantly different across the three periods. Similar ANOVA tests were then run for annual caseload and revenue statistics (outpatient visits, inpatient discharges, annual revenue and average salary) and service capacity statistics (total asset value, facility’s square footage, number of beds, and number of medical devices with purchase price higher than 10,000 Yuan).

As to qualitative data, two members of our team listened to the records of focus group again. They collected the key words that informants mentioned frequently.

## Results

The characteristics of township-village health centers, central township health centers and county hospitals were described and compared in Table 1. We can see that each township-village health centers had an average of 40 staff members before the privatization, and majority of those staff members were their permanent staff. On average, there were 22 doctors and 7 nurses in one health center prior to the privatization. In 2005, three years after the privatization, a noticeable amount of decrease (from 39.7 staff members per TVHC to 27.5 per TVHC) occurred in these township-village health centers. From 2000 to 2009, the total payroll in TVHCs decreased by almost 29%, while the number of their permanent staff members and nurses decreased by more than 40%. Central township health centers had no significant change on their payroll size during this period, although there had been a sizable decrease in permanent staff members and nurses. At a time when privatized TVHCs lost their staff members, county hospitals’ average payroll size increased by 20%, especially for the number of doctors.

**Table 1:** Human resources among health care organizations in Haimen

**Items**	**Township-village health center (n = 25)**					**Central health center (n = 6)**					**County hospital (n = 3)**				
	**Mean**	**Standard error**	**95% CI for mean**		**P value (ANOVA)**	**Mean**	**Standard error**	**95% CI for mean**		**P value (ANOVA)**	**Mean**	**Standard error**	**95% CI for mean**		**P value (ANOVA)**
			**Lower**	**Upper**				**Lower**	**Upper**				**Lower**	**Upper**	
Staff per organization					**0.000**					0.997					0.960
2000	39.7	1.7	36.3	43.2		153.3	22.9	94.6	212.1		414.7	158.7	−268.4	1097.7	
2005	27.5	1.7	24.2	31.0		155.2	29.9	78.3	232.0		471.0	220.3	−476.7	1418.7	
2009	28.2	2.8	22.5	33.9		152.2	34.7	63.0	241.4		496.0	233.4	−508.3	1500.3	
Permanent staff per organization					**0.000**					0.661					0.989
2000	39.7	1.7	36.2	43.1		153.0	23.1	93.7	212.3		380.7	155.8	−289.7	1051.1	
2005	26.9	1.4	24.0	30.0		137.3	22.3	80.1	194.6		407.0	180.8	−371.0	1185.0	
2009	21.9	1.4	19.1	24.7		123.3	22.9	64.6	182.1		417.3	194.0	−417.4	1252.0	
Doctors per organization					**0.001**					0.794					0.975
2000	21.6	1.0	19.5	23.7		54.3	5.9	39.1	69.6		142.3	63.9	−132.5	417.2	
2005	17.2	1.0	15.1	19.2		47.0	6.0	31.6	62.5		151.3	75.0	−171.2	473.9	
2009	16.7	0.8	15.1	18.4		54.3	12.6	21.9	86.7		166.0	84.0	−195.4	527.4	
Nurses per organization					**0.001**					0.096					0.992
2000	7.1	0.5	6.1	8.1		32.2	4.9	19.5	44.8		110.7	52.9	−117.0	338.4	
2005	5.4	0.7	4.0	6.8		27.2	4.2	16.4	37.9		118.0	62.6	−151.5	387.5	
2009	4.1	0.4	3.3	4.9		18.3	3.4	9.5	27.2		121.7	66.3	−163.5	406.9	
% permanent staff	Percentage					Percentage					Percentage				
2000	100.00%					99.80%					91.80%				
2005	97.82%					88.47%					86.41%				
2009	77.66%					81.01%					84.13%				

In 2000, the average salary was ¥17,400 in TVHCs and decreased to ¥15,000 in 2009 after adjusting for inflation. CTHC had the similar level of salary as TVHCs in 2000, however their salary level reached nearly ¥27,000 per person in 2009 (76% higher than health TVHCs). County hospitals paid ¥20,000 per person in 2000, higher than that of TVHCs and central health centers. By 2009 the salary among county hospitals increased by about 80%, faster than the rate of increase among central health centers (see Table 2).

**Table 2:** Annual caseload and revenue statistics of health care providers in Haimen

**Items**	**Township-village health center (n = 25)**					**Central health center (n = 6)**					**County hospital (n = 3)**				
	**Mean**	**Standard error**	**95% CI for mean**		**P value (ANOVA)**	**Mean**	**Standard error**	**95% CI for mean**		**P value (ANOVA)**	**Mean**	**Standard error**	**95% CI for mean**		**P value (ANOVA)**
			**Lower**	**Upper**				**Lower**	**Upper**				**Lower**	**Upper**	
Number of outpatient visits (thousands)					0.158					0.270					0.382
2000	19.3	1.3	16.6	22.0		29.4	7.8	21.2	37.6		105.8	36.1	−49.7	261.3	
2005	15.9	1.3	13.3	18.5		26.5	8.0	18.1	35.0		152.4	42.5	−30.6	335.4	
2009	17.8	1.2	15.3	20.4		40.6	23.8	15.6	65.5		236.8	92.3	−160.3	633.8	
Number of discharges					0.338					0.166					0.576
(thousands)															
2000	0.5	0.0	0.4	0.6		1.6	0.2	1.0	2.2		5.2	2.9	−7.1	17.5	
2005	0.5	0.1	0.4	0.6		1.8	0.4	0.9	2.8		8.5	4.9	−12.6	29.6	
2009	0.6	0.1	0.5	0.8		2.8	0.7	1.2	4.5		13.4	7.1	−17.2	43.9	
Annual revenue					0.661					0.143					0.484
(million yuan) *															
2000	2.0	0.1	1.7	2.3		6.8	1.2	3.6	9.9		28.2	15.0	−36.4	92.9	
2005	2.3	0.2	1.8	2.7		9.7	2.3	3.7	15.7		69.8	42.2	−111.6	251.2	
2009	2.1	0.2	1.7	2.6		15.8	4.7	3.8	27.8		115.3	70.1	−186.5	417.1	
Average salary					0.102					**0.000**					0.111
(ten thousand)*															
2000	1.7	0.0	1.6	1.8		1.7	0.0	1.6	1.8		2.0	0.3	0.5	3.4	
2005	1.6	0.1	1.4	1.7		2.1	0.0	2.0	2.2		2.6	0.5	0.6	4.6	
2009	1.5	0.1	1.3	1.7		2.7	0.2	2.2	3.2		3.5	0.4	1.6	5.3	

Notes:

*: All the data were adjusted to 2000 by CPI.

In terms of capital accumulation, an average TVHC had a total asset value of ¥2.73 million in 2000, and increased almost 13% to 3 million during next 10 years, adjusting for inflation. However the average total asset value in central health centers and county hospitals had increased near to 2 times and 3.6 times respectively since 2000 (Table 3). Table 3 also compares the trends in caseload among three different types of health care providers: TVHCs provided 19000 outpatient cases and 500 inpatient cases in 2000, and experienced almost no changes in caseload after the privatization, while the two other types of providers both experienced substantial increase in inpatient and outpatient caseload. These different trends in health service capacity are also reflected in different trends in annual revenue: the annual revenue of TVHCs showed no obvious increase after the privatization, whereas the annual revenue of central health centers increased by 30% and the average annual revenue of county hospitals tripled since 2000 (Table 2).

**Table 3:** Asset value and service capacity among health care providers in Haimen

**Items**	**Township-village health center (n = 25)**					**Central health center (n = 6)**					**County hospital (n = 3)**				
	**Mean**	**Standard error**	**95% CI for mean**		**P value (ANOVA)**	**Mean**	**Standard error**	**95% CI for mean**		**P value (ANOVA)**	**Mean**	**Standard error**	**95% CI for mean**		**P value (ANOVA)**
			**Lower**	**Upper**				**Lower**	**Upper**				**Lower**	**Upper**	
Number of beds					0.545					0.714					0.759
2000*															
2005	32.4	1.6	29.2	35.6		99.2	19.3	49.6	148.8		237.0	101.6	−200.16	674.2	
2009	33.8	1.7	30.3	37.3		110.8	24.2	48.7	173.0		282.0	91.9	−113.4	677.4	
Number of medical devices					0.226					0.213					0.548
(over 10 thousand Yuan)															
2000	6.4	0.9	4.6	8.1		31.5	3.4	22.9	40.2		106.3	51.4	−114.6	327.3	
2005	7.2	0.8	5.5	8.8		52.8	11.7	22.6	83.0		206.0	99.1	−220.5	632.5	
2009	8.5	1.0	6.5	10.5		54.8	11.9	24.1	85.5		285.7	154.6	−379.3	950.6	
Square footage					0.667					0.479					0.637
(thousand m^2^)															
2000	3.4	0.3	2.8	4.0		6.8	1.3	3.4	10.3		14.0	5.4	−9.27	37.2	
2005	3.7	0.3	3.2	4.3		9.1	1.6	5.1	13.2		25.8	11.2	−22.3	74.0	
2009	3.6	0.2	3.2	4.1		9.0	1.5	5.2	12.8		27.4	13.3	−29.8	84.6	
Total asset value					0.717					0.191					0.507
(million Yuan)**															
2000	2.7	0.3	2.2	3.3		8.4	1.0	5.7	11.1		32.1	14.7	−31.3	95.5	
2005	3.1	0.4	2.3	3.8		15.0	2.7	8.1	22.0		100.3	60.2	−158.8	359.4	
2009	3.1	0.4	2.3	3.8		24.3	9.7	−0.8	49.4		146.4	95.9	−266.0	558.9	

Notes:

*: We hadn’t the data of bed count in 2000.

**: All the data were adjusted to 2000 by CPI.

These results seem to fit what we heard during the focus groups. Although the total caseload hasn’t increased after privatization, the remaining staff members had to work for extended hours and to increase workload per person due to the reduced number of colleagues. This was often mentioned during the focus groups as one of the major changes the 2002 privatization had brought to TVHCs. A manager from a TVHC mentioned that their in-house pharmacy currently accounted for more than 90% of the total revenue, indicating a lack of cash inflow for the health care provider.

## Discussion

According to our findings, the number of permanent staff members in privatized health centers decreased sharply after ownership reform, a finding that strengthens several previous observations with our longitudinal data [28-30]. There are two possible reasons to explain this phenomenon. One is the financial pressure forced these privatized health centers to downsize their payroll [31-33]. Prior to the ownership reform, township health centers were not very profitable and thus depended upon governmental subsidy for their very survival [34]. After privatization, those privatized health centers no longer received any financial resource from local governments. Moreover, the average rate of revenue increase in privatized health centers from 2000 to 2009 was only one twentieth of that in unprivatized health centers (Table 1). The result could be that the revenue in these privatized health centers may not offset the increasing operation cost, and thus they had to cut down on their payrolls as one way of cost containment.

The other explanation is that employees in privatized health centers might choose to leave partly due to undesirable career prospects and financial insecurity. China’s medical professionals in public hospitals heavily rely on their employers for pension, health insurance, further education or training, housing, etc. In a way, they are an alternative kind of government employees. After privatization, these professionals live from contract to contract, which means they lose the stable benefits they used to have. And this loss in benefits has not been compensated for by higher salary. Instead, our finding showed that the average per person salary in privatized health centers actually declined after the privatization during a time when the country’s GDP per capita almost tripled. According to our focus group discussions, the performance evaluation in the privatized township health centers was entirely based on their physicians’ caseload, whereby a higher caseload per physician brings in higher salary for physicians. However, local residents would prefer to go to county hospitals if they have better insurance coverage or have relatively high income, while physicians in township health centers do not have the authority of “gate-keeper” to sign off a visit to county hospitals [35]. The low-income patients and patients covered by the more rudimentary Rural Cooperative Medical System (RCMS) [36] might be more likely to use township-village level facilities. And many of these potential patients might be more likely to visit central township health centers, which still had public ownership [37]. Therefore the privatized health centers are only left with the low-income patients whose insurance coverage were not that generous at a time they lost the government subsidy, contributing to their low revenue and salary level. For the elder staff members, their pension might still be an incentive for retention, while younger staff members could find it more optimal to resign and seek their career elsewhere. Studies showed that staff members in township health centers were more likely to oppose ownership reform than leaders of health centers and officers in local government [38]. It is no surprise then why we witnessed the loss of human resources after the privatization.

However, we have to admit that there exists an alternative hypothesis that could explain the unsuccessful staff retention among township health centers: China’s urbanization trend. The rural population in Jiangsu Province has been shrinking since 2000 at an average annual rate of 2.6% [39], a common phenomenon throughout China. So the demand for health service at the township level has been lower than before. This may be also an important reason to explain the unsuccessful staff retention in township health centers. In our study, we didn’t have enough data to rule out this possible causal mechanism.

Ownership reform in China in health system is a reform initiated by the central government [40]. The aim was to attract the individual or organizational capital toward the health system, and then relieve the heavy financial distress on local government [41]. However, from the statistics we collected, the development of privatized health centers seriously lagged behind their counterparts in the public sector, in financial resources and human resources alike. This is not an optimal scenario as township health centers provide fundamental health services to most residents in rural area, who are either without insurance or covered by the rudimentary RCMS. To some extent, township health centers in China share some functions with the local government in term of providing uncompensated care [42]. So the ownership reform in township health centers should be different from that in comprehensive teaching hospitals in urban areas, as those hospitals have a much richer client pool to serve. There were evidences that the government could help more with the health institution’s ownership reform [40]. One suggestion for the local government is to be a sustainable payer to buy these private health services at the township level. As in Turkey, government agencies purchase some of their services from private hospitals, which become a substantial source of revenue for private hospitals [43]. The other suggestion is to pay physicians by the number of insured people, not by the number of patient visits. As in UK, although general practitioners are entirely in the private sector, they still have received public pensions funded jointly with state [44]. For ownership reform among township-level health care facilities, local government should also monitor the post-privatization development and help foster a better policy environment for private health centers (e.g., tax breaks for for-profit private hospitals and tax deduction for donations to nonprofit private hospitals) if unwanted consequence in staff retention is witnessed.

Several important limitations compromise the external validity of our study. Firstly, we didn’t adjust staff ratios based on case-mix index, since there hadn’t been official index published in China by the time we wrote this article. Commonly, health services provided by township health centers are unitary and simple. In addition, all health centers in our study located in same city. So health services provided in these facilities are almost consistent. Comparing the staff amount in different health centers directly wouldn’t significantly bias the result. Secondly, although we briefly interviewed health center executives for a better understanding of the background information, we didn’t interview those staff members who left health centers during or after ownership reform. This would limit our understanding of the real reasons behind their resignation.

## Conclusions

As our case showed, one of the unwanted results of the privatization could be that the total amount of health professionals decreased. Without adequate and sustained support for these health professionals working at the frontline of China’s health, township health centers after privatization are experiencing a serious brain drainage, which threatens the very base of China’s population health. The local government, simply by strengthening the social safety net for all working people, can substantially reduce the career risk of those who work in privatized health centers and thus make those health centers competitive in providing primary health care for the rural communities. In the short-term, stronger financing for the rural health insurance plan (New Rural Cooperative Scheme) might be an efficient and effective way of improving the financial well-being of privatized TVHCs.

## Competing interests

The authors declare that they have no competing interests.

## Authors’ contributions

JH participated in the design of the study, performed the statistical analysis, and prepared the initial draft of the manuscript. LS contributed to the writing of all five sections. YC designed and coordinated the study and assisted in preparing the manuscript. All authors read and approved the final draft of the manuscript.

## Pre-publication history

The pre-publication history for this paper can be accessed here:

http://www.biomedcentral.com/1472-6963/13/136/prepub
